# Supplementary material for: Model retraining and information sharing in a supply chain with long-term fluctuating demands
Source: Sci Rep. 2021 Oct 13;11:20277. doi: 10.1038/s41598-021-99542-z (PMC8514521; doi:10.1038/s41598-021-99542-z)
Supplement: Supplementary file 1 — Supplementary Information. [file 41598_2021_99542_MOESM1_ESM.pdf]

Supporting Information for: Model retraining and information sharing in a supply chain with long-term fluctuating demands

Takahiro Ezaki*, Naoto Imura, Katsuhiro Nishinari

September 20, 2021

Demands following a log-normal distribution

In the main text, we assumed that the demands follow the Gaussian distribution. Here, to confirm that our conclusions are not sensitively influenced by the choice of the probability distribution, we performed simulations with the following log-normal distribution:

$$O_0 \sim \frac{1}{\sqrt{2\pi}\sigma_0 O_0} \exp\left[-\frac{(\ln O_0 - \mu_0)}{2\sigma_0^2}\right]. \tag{S1}$$

With this distribution, the logarithm of O_0 follows the Gaussian distribution, i.e., $\ln O_0 \sim \mathcal{N}(\mu_0, \sigma_0)$. The mean and variance of this distribution for fixed μ_0 and σ_0, are expressed as $E[O_0 \mid \mu_0, \sigma_0] = \exp[\mu_0 + \sigma_0^2/2]$ and $V[O_0 \mid \mu_0, \sigma_0] = \exp[2\mu_0 + \sigma_0^2](\exp[\sigma_0^2] - 1)$, respectively.

To implement the constant policy for this distribution, we numerically computed the expected value of O_0 (with varying $\mu_0(\sim U(0.5, 2))$) to obtain $\bar{\mu} \approx 3.845$. This value was used to normalize the lost sales opportunities as well. The other settings of simulations were left unchanged from those used in the main text.

The results are shown in Supporting Figs. 1–3. Because the mean and variance of the demands are larger than those used in the main text, the average inventory level and percentage of lost sales opportunities were larger than the results reported in Figs. 2–4 in the main text. However qualitative characteristics of the results were very similar, suggesting the robustness of our conclusions.

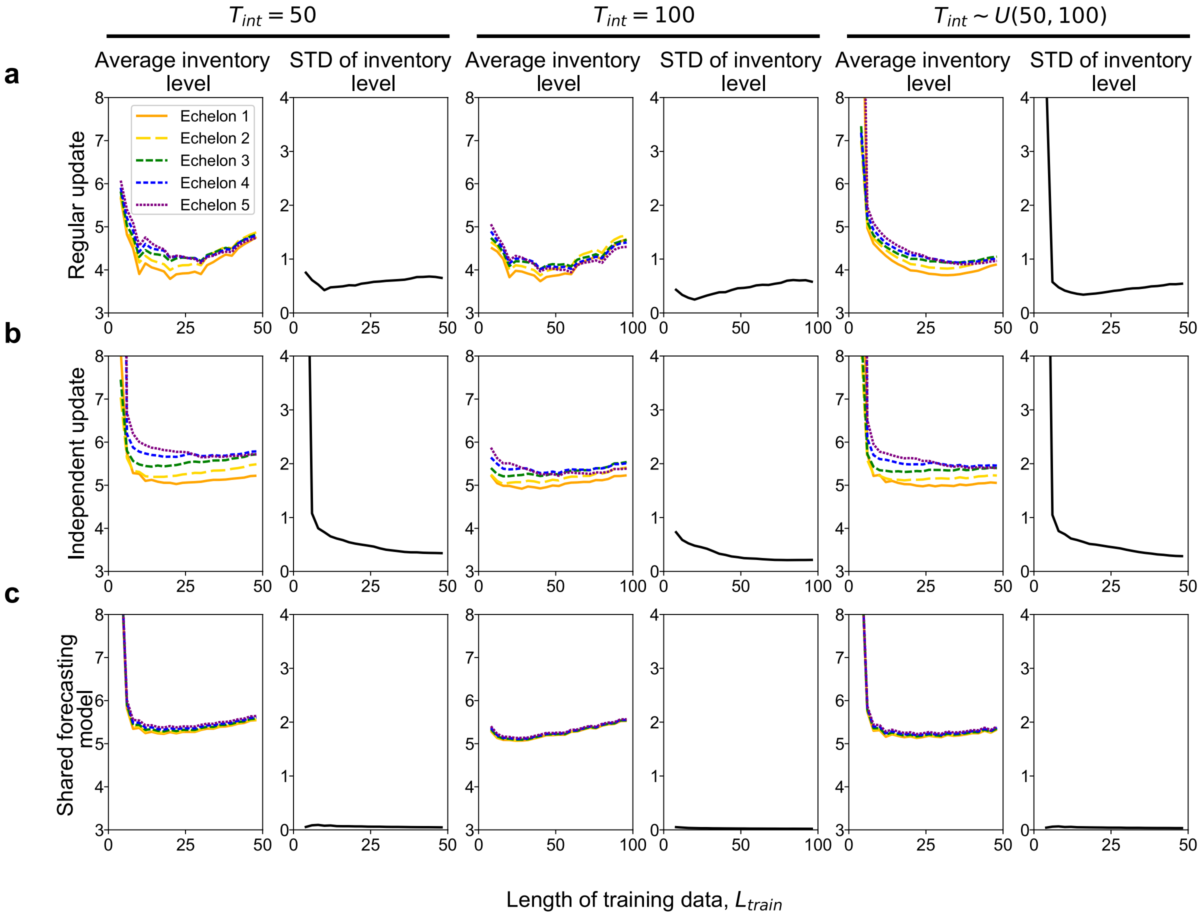

Supplementary Figure 1. Average inventory level at each echelon. (a) Regular update scheme. (b) Independent update scheme. (c) Shared forecasting model scheme. In each panel, we varied the length of training data, L_{train}. Simulations were performed for three types of intervals of demand change: $T_{\text{int}} = 50$ (left), $T_{\text{int}} = 100$ (middle), and $T_{\text{int}} \sim U(50, 100)$ (right). The standard deviation of the inventory level for 5 echelons was computed for each time step. For each simulation condition, the results were averaged over $t = 10^7$ steps.

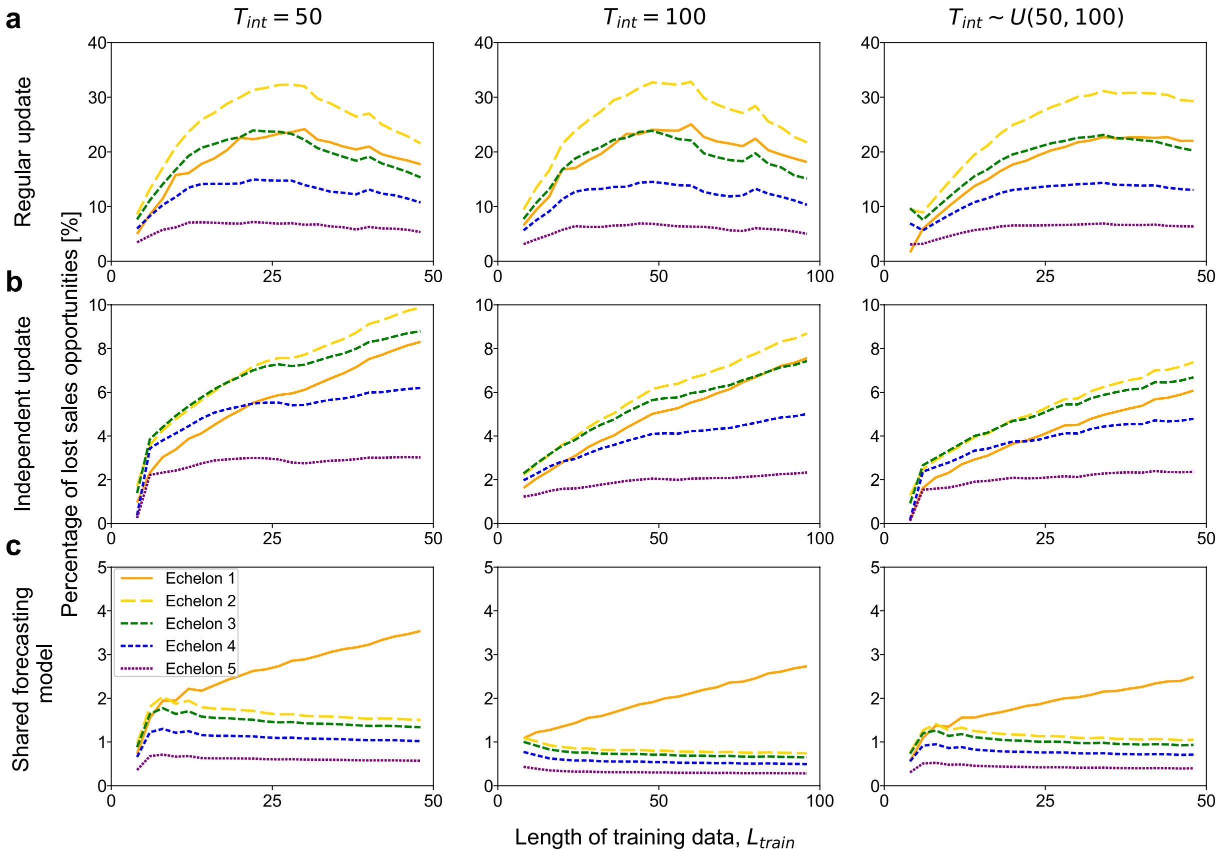

Supplementary Figure 2. Percentage of lost sales opportunities. (a) Regular update scheme. (b) Independent update scheme. (c) Shared forecasting model scheme. In each panel, we varied the length of training data, L_{train}. The simulations were performed for three types of intervals of demand change, i.e., $T_{\text{int}} = 50$ (left), $T_{\text{int}} = 100$ (middle), and $T_{\text{int}} \sim U(50, 100)$ (right). For each simulation condition, the results were averaged over $t = 10^7$ steps.

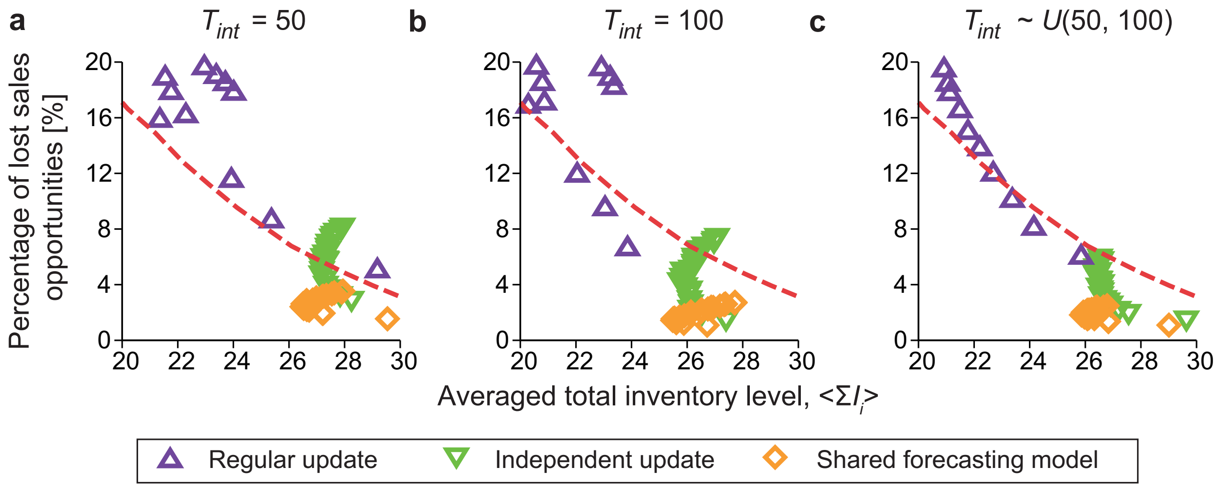

Supplementary Figure 3. Trade-off between the average inventory level (Supplementary Fig. 1) and the percentage of lost sales opportunities (Supplementary Fig. 2). (a) $T_{\text{int}} = 50$. (b) $T_{\text{int}} = 100$. (c) $T_{\text{int}} \sim U(50, 100)$. Each symbol represents a simulation condition (i.e., a single L_{train} value) in Supplementary Figs. 1 and 2. The red dashed line represents the results of the constant policy.
